# Years of palpitations and a heart rate of 213 beats per minute

**DOI:** 10.1002/ccr3.1021

**Published:** 2017-06-02

**Authors:** Kevin Lee, Joseph Banta, Matthew D'Ambrosio, Apostolos Voudouris, Antonios Tsompanidis

**Affiliations:** ^1^ Rowan University School of Osteopathic MedicineStratfordNew Jersey08084; ^2^ Department of CardiologyCarePoint Health Christ HospitalJersey CityNew Jersey07306; ^3^ Family Medicine Residency Program Director/DMECarePoint Health Christ HospitalJersey CityNew Jersey07306

**Keywords:** Belhassen tachycardia, bundle branch block, re‐entrant circuit, verapamil

## Abstract

Belhassen tachycardia is the most common idiopathic ventricular tachycardia arising from the left ventricle, classically characterized by a right bundle branch block and left axis deviation. Vigilance for Belhassen tachycardia is essential as intravenous verapamil has proven to be highly efficacious for treating symptomatic patients with this underlying arrhythmia.

## Case Presentation

**Figure 1:**
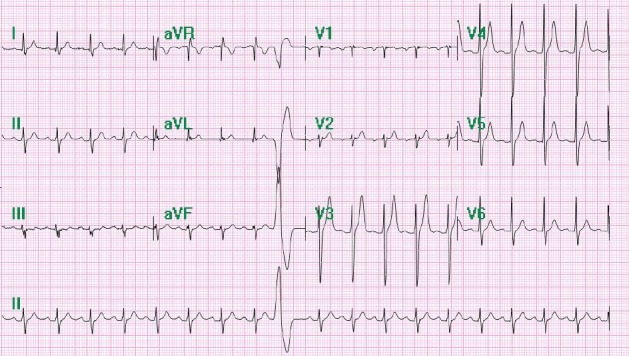
Initial ECG upon emergency department evaluation showing sinus tachycardia with occasional premature ventricular complexes.

**Figure 2:**
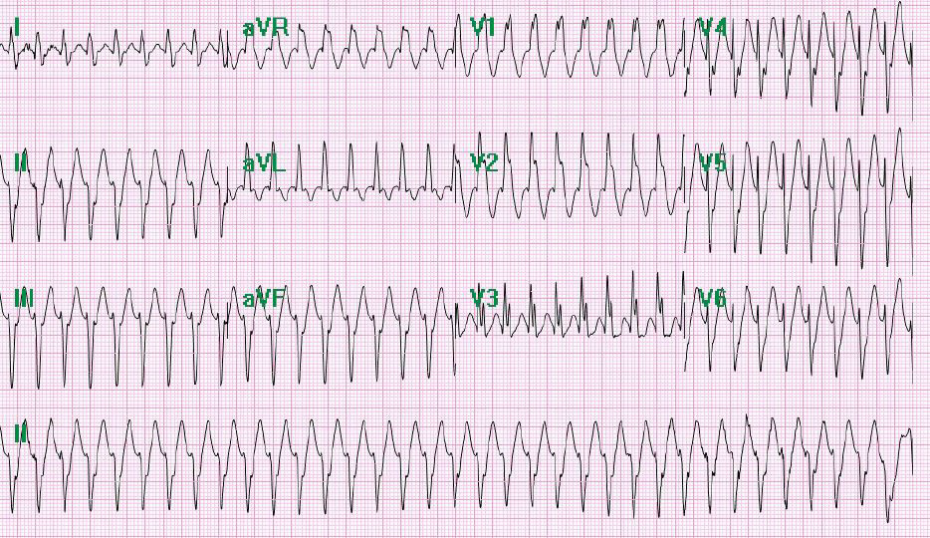
Repeat ECG in the emergency department indicating Belhassen tachycardia as evidenced by a rate of 213 bpm and a right bundle branch block (RBBB) and left axis deviation. This was a unique case of Belhassen ventricular tachycardia in which the heart rate greater than 200 bpm and a QRS that intermittently dropped below 120 msec were seen.

**Figure 3:**
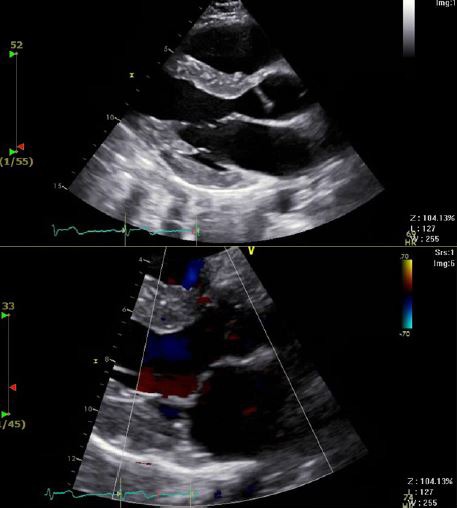
Echocardiogram findings indicated no significant structural heart disease. Mild tricuspid regurgitation, mild mitral regurgitation, and normal left ventricle systolic function with an ejection fraction of 60–65% were all noted.

A 39‐year‐old Hispanic Male with no past medical history presented to the emergency department with palpitations and a rapid heart rate. His symptomology was associated with lightheadedness and chest pain radiating to his left shoulder. He remained hemodynamically stable and coherent throughout his hospital course. The patient had experienced palpations for years, but previous evaluations demonstrated an inconclusive etiology. As part of initial management, the standard 12‐lead electrocardiogram (ECG) was obtained which revealed sinus tachycardia with occasional premature ventricular complexes (Fig. 1). A repeat ECG 2 h later showed a heart rate of 213 bpm and a right bundle branch block (RBBB) with left axis deviation, suggesting a phenomenon from the left ventricle (Fig. 2). Echocardiogram showed mild tricuspid regurgitation, mild mitral regurgitation, and normal left ventricle systolic function with an ejection fraction of 60–65%, ruling out any significant structural defects (Fig. 3).

Question: What is the likely electrophysiology which contributed to the ECG findings and symptomology?

- Re‐entrant circuit arising from the posterior fascicle and extending apically
- Decreased AV conduction associated with digoxin toxicity
- Ventricular tachycardia originating from the right ventricle
- Spontaneous transition from premature atrial complexes to atrial fibrillation

Answer: (A) Re‐entrant circuit arising from posterior fascicle and extending apically.

The ECG findings suggest a morphology associated with an idiopathic QRS Belhassen tachycardia. Given this finding, this arrhythmia was likely due to an ectopic focus within the left ventricle causing a re‐entrant tachycardia arising from the posterior fascicle and extending apically along the inferoposterior septum in an anterograde fashion, Choice A 1. As the cardiac conduction system generates subsequent electrical impulses, standard ventricular activation from the Purkinje fibers to the ventricles is no longer preserved as a consequence of the aforementioned ectopic focus. It is believed that this re‐entry tachycardia stems from abnormal Purkinje fibers because of its dependence on the slow conduction of calcium in partial depolarization 2. This is significant because Purkinje fibers are targeted and guide therapy in acute management of Belhassen tachycardia through the use of verapamil. It is known that idiopathic ventricular tachycardias depend on slow entry calcium in partially depolarized Purkinje fibers, thus making verapamil the ideal first‐line treatment. 12 mg of IV Adenosine was used initially, but the arrhythmia was refractory to such treatment allowing us to confirm our diagnosis of fascicular tachycardia. It should be emphasized that Belhassen tachycardia is refractory to beta blockers, adenosine, and vagal maneuvers because its pathway is not cAMP mediated, unlike other adenosine‐sensitive ventricular tachycardias.

Treatment involved chemical cardioversion with intravenous verapamil for the patient's incessant paroxysmal tachycardia. His heart rate of 213 beats per minute was concerning as this was not only an extremely rapid heart rate that contributed to his initial symptomology, but was also a very rare phenomenon in fascicular ventricular tachycardias. Catheter ablation was offered, but the patient opted for chronic long‐term oral verapamil and follow‐up in the clinic. This case highlights an atypical presentation of Belhassen ventricular tachycardia in which a heart rate greater than 200 bpm and a QRS that intermittently dropped below 120 msec were seen 3.

## Conflicts of Interest

The authors do not have conflicts of interest to disclose. No material support was provided for this article.

## Authorship

KL: Wrote and drafted the manuscript. Addressed comments by editor after two revisions. Discussed the importance of ECG for educational merit with Dr. Voudouris. JB: Edited manuscript for accuracy and completeness. Assisted with literature search. Helped improve resolution of images. Discussed the importance of ECG for educational merit with Dr. Voudouris. MD: Edited manuscript for accuracy and completeness. Assisted with literature search. Helped improve resolution of images. Discussed the importance of ECG for educational merit with Dr. Voudouris. AV: Managed patient and presented ECG findings during cardiology rotation. Highlighted electrophysiology and rarity of patient's presentation. AT: Reviewed and approved manuscript as per CarePoint Health Christ Hospital's patient safety and policies. Research advisor and provided guidance in submission. Provided insight on electrophysiology.
